# *Piper fuligineum* (Piperaceae) Essential Oil on *Euschistus heros*: Lethal and Sublethal Toxicities and Selectivity to an Egg Parasitoid Used in Applied Biological Control

**DOI:** 10.3390/molecules31213988

**Published:** 2026-11-05

**Authors:** Vanessa Cardoso Nunes, Jonathan Pereira, Diones Krinski, Daniela Hartwig de Oliveira, Michele Trombin de Souza, Mireli Trombin de Souza, Leandro do Prado Ribeiro, Odimar Zanuzo Zanardi, Daniel Bernardi, Anderson Dionei Grutzmacher

**Affiliations:** 1 Department of Plant Health, Federal University of Pelotas, Capão-do-Leão 96010-900, Rio Grande do Sul, Brazil; cardoso.n.vanessa@gmail.com (V.C.N.); jonathan.agronomo@gmail.com (J.P.); micheletrombin@gmail.com (M.T.d.S.); mirelitrombin@gmail.com (M.T.d.S.); adgrutzm@ufpel.edu.br (A.D.G.); 2 Department of Biological Sciences, Mato Grosso State University, Tangará da Serra 78300-000, Mato Grosso, Brazil; diones.krinski@unemat.br; 3 Department of Chemical, Pharmaceutical, and Food Sciences, Federal University of Pelotas, Capão-do-Leão 96010-900, Rio Grande do Sul, Brazil; doliveira.iqg@ufpel.edu.br; 4 Research Center for Family Agriculture, Agricultural Research and Rural Extension Company of Santa Catarina (CEPAF/EPAGRI), Chapecó 89801-970, Santa Catarina, Brazil; leandroribeiro@epagri.sc.gov.br; 5 Department of Education, Research, and Extension, Federal Institute of Santa Catarina, São Miguel do Oeste 89900-000, Santa Catarina, Brazil; odimarzanardi@yahoo.com.br

**Keywords:** botanical insecticide, ovicidal activity, stage-dependent toxicity, parasitoid selectivity

## Abstract

*Euschistus heros* is a major soybean pest in Neotropics, requiring sustainable control alternatives. This study characterized the chemical composition of *Piper fuligineum* essential oil (EOPF) and evaluated its bioactivity against different developmental stages of *E. heros*, as well as its selectivity toward the egg parasitoid *Telenomus podisi*. Chemical analysis identified 19 compounds accounting for 85.37% of EOPF, with a profile dominated by sesquiterpenes, particularly β-caryophyllene (10.60%), β-eremophyllene (10.46%), and eremophyllene (9.09). The major monoterpenes were 1-naphthalenol (9.88%), β-ocimene (5.48%), and β-pinene (5.16%). EOPF exhibited pronounced stage-dependent ovicidal activity, causing up to 91% egg inviability during early embryogenesis at the highest concentration tested (40,000 mg L^−1^). Exposure also markedly reduced the subsequent survival of nymphs emerging from treated eggs and caused concentration-dependent mortality in postembryonic stages, reaching, at 40,000 mg L^−1^, 53% in third-instar nymphs and 46% in adults. Selectivity toward *T. podisi* strongly depended on the timing of exposure. In pre-parasitism assays, parasitism and emergence were reduced to near 0% at ≥10,000 mg L^−1^, while in post-parasitism conditions, parasitism remained high (91–95%) but emergence declined to 0% at the highest concentration. Free-choice tests showed a pronounced repellent effect of EOPF, with females preferentially parasitizing untreated eggs. According to the IOBC classification, the EOPF ranged from slight to harmful, particularly affecting parasitoid emergence. These findings highlight the need to optimize concentration, exposure timing, and application strategy before integrating EOPF with parasitoid-based biological control.

## 1. Introduction

The soybean brown stink bug, *Euschistus heros* Fabricius (Hemiptera: Pentatomidae), is one of the most economically important pests of soybean in the Neotropical region, particularly in Brazil and neighboring countries [1,2]. Feeding by nymphs and adults causes direct damage to developing pods and seeds, leading to reductions in yield and seed quality, as well as physiological disorders that make harvesting difficult [3,4]. The widespread adoption of intensive soybean production systems with successive cropping has favored population outbreaks of *E. heros* by conditioning the “green bridge”, increasing reliance on chemical insecticides as the primary control strategy [5,6]. However, the limited number of available active ingredients, as well as their overuse, has resulted in the selection of resistant populations and adverse environmental impacts, including the disruption of natural enemies and their ecological services, reinforcing the urgent need for sustainable approaches within an Integrated Pest Management (IPM) approach [7,8].

Among alternative strategies, biological control using egg parasitoids such as *Telenomus podisi* Ashmead (Hymenoptera: Scelionidae) plays a pivotal role in the natural regulation of pentatomid populations [9,10,11]. This parasitoid is widely distributed in soybean-growing regions and can parasitize a substantial proportion of stink bug egg masses under field conditions [12]. By attacking the pest at the egg stage, *T. podisi* prevents crop injury before nymphal feeding begins, making it a valuable component of IPM strategies [13]. Nevertheless, the compatibility of biological control agents with other management tactics, particularly botanical insecticides, must be carefully evaluated to ensure selectivity and conservation of beneficial arthropods.

Plant-derived essential oils (EOs) have gained attention as promising alternatives to synthetic insecticides due to their complex chemical composition, rapid environmental degradation, and diverse modes of action. Species of the genus Piper (Piperaceae) are known for producing bioactive secondary metabolites (allelochemicals) with insecticidal, repellent, and growth-disrupting properties [14,15]. In this context, *Piper fuligineum* Kunth has emerged as a potential source of bioactive compounds [16]; however, its effects on *E. heros* and on associated natural enemies remain poorly understood. Assessing both efficacy against the target pest and selectivity toward key parasitoids is essential for determining the feasibility of incorporating such botanical products into IPM programs.

Given this context, the present study tested the hypotheses that the EO of P. fuligineum (EOPF) exhibits (1) promising lethal and sublethal effects on different stages of development of *E. heros* and potential to be applied in the framework of the soybean stink bug complex, and (2) selectivity to an egg parasitoid widely used in applied biological control programs. To test these hypotheses, bioassays were conducted to (i) determine the susceptibility of *E. heros* eggs to the across embryonic stages; (ii) evaluate the effects of sublethal exposure to the EOPF on the survival, development, and behavior of newly hatched nymphs; (iii) assess the toxicity of the EOPF to nymphal and adult stages of *E. heros*; and (iv) examine its selectivity toward *T. podisi*, focusing on parasitism, development, and emergence under pre- and post-parasitism exposure conditions. These findings contribute to ongoing efforts to generate research-based evidence on the efficacy of newly derived botanical insecticides and their compatibility with natural enemies commonly present in agroecosystems, thereby supporting the advancement of more environmentally sustainable pest management practices.

## 2. Results

In the chemical analysis of the EOPF, 19 compounds were identified, accounting for 85.37% of the total composition. Seven major compounds were detected: caryophyllene (10.60%), β-eudesmene (10.46%), 1-naphthalenol (9.88%), eremophyllene (9.09%), germacrene D (7.40%), β-ocimene (5.48%), and β-pinene (5.16%). Together, these compounds represented 58.07% of the EOPF composition, each occurring at concentrations above 5%. The remaining 12 compounds were present in lower concentrations, ranging from 1.29% to 4.41%, and collectively accounted for 27.30% of the total EOPF composition (Table 1).

The EOPF significantly reduced *E. heros* egg hatching in a concentration-dependent manner at all egg ages (Figure 1). All fitted concentration–response models were significant (R2 = 0.6596–0.8044; *p* ≤ 0.0006). The highest model fit was observed for 1-day-old (R2 = 0.7350; Figure 1A), 2-day-old-eggs (R2 = 0.8044; Figure 1B), followed by 3-day-old (R2 = 0.7482; Figure 1C). The magnitude of the ovicidal effect decreased with embryonic age, with younger eggs showing a sharper reduction in hatching as EOPF concentration increased. At the highest concentration, hatching was reduced to approximately 10–20% in 1-to-3-day-old eggs, whereas approximately 30% of 4- and 5-day-old eggs still hatched. These results indicate that advanced embryonic development reduced egg susceptibility to EOPF. Engeo Pleno^TM^ S, used as the positive control, caused nearly complete inhibition of nymph hatching at all egg ages.

The EOPF significantly increased mortality of third- and fifth-instar *E. heros* nymphs, as well as adults, as a function of concentration (Figure 2). The fitted models were significant for third-instar (R2 = 0.6882; F = 149.638; *p* = 0.0058; Figure 2A), fifth-instar nymphs (R2 = 0.6931; F = 105.034; *p* = 0.0054; Figure 2B), and adults (R2 = 0.6451; F = 89.177; *p* = 0.0078; Figure 2C). Mortality increased more sharply at lower concentrations and subsequently approached a plateau, reaching approximately 50% for third-instar nymphs, 40% for fifth-instar nymphs, and 45% for adults. Among the evaluated developmental stages, third-instar nymphs showed the highest response to EOPF, whereas fifth-instar nymphs exhibited the lowest response. Engeo Pleno^TM^ S, used as the positive control, resulted in approximately 95–100% mortality across all developmental stages.

Lethal concentration estimates revealed clear stage-dependent differences in the susceptibility of *E. heros* to EOPF (Table 2). Among eggs, LC_50_ values increased from 1100 mg L^−1^ in 1-day-old eggs to 2000 mg L^−1^ in 5-day-old eggs, whereas LC_90_ values ranged from 4700 mg L^−1^ to 13,600 mg L^−1^. Therefore, lower EOPF concentrations were required to cause mortality in younger eggs, confirming their susceptibility during early embryogenesis. Among postembryonic stages, third-instar nymphs were the most susceptible (LC_50_ = 4000 mg L^−1^; LC_90_ = 20,000 mg L^−1^), followed by adults (LC_50_ = 6200 mg L^−1^; LC_90_ = 30,900 mg L^−1^). Fifth-instar nymphs were the least susceptible stage, exhibiting the highest LC_50_ (9700 mg L^−1^) and LC_90_ (54,800 mg L^−1^) values.

The effects of EOPF on *T. podisi* were dependent on the timing of exposure (Figure 3). In the pre-parasitism bioassay, increasing EOPF concentration significantly reduced both parasitism and parasitoid emergence. Parasitism declined sharply at the lower concentrations and subsequently approached a plateau of approximately 14% at higher concentrations (Figure 3A). The fitted model was significant (R2 = 0.7586; F = 179.975; *p* < 0.0001). Parasitoid emergence was even more affected, decreasing rapidly with increasing EOPF concentration and approaching zero at concentrations ≥ 10,000 mg L^−1^ (Figure 3B). In the post-parasitism bioassay, parasitism was comparatively less affected by EOPF exposure (Figure 3C). Although a significant concentration-dependent response was detected (R2 = 0.5533; F = 91.451; *p* = 0.0397), parasitism remained relatively high across the concentration range and approached a plateau of approximately 75%. In contrast, parasitoid emergence declined sharply with increasing EOPF concentration (Figure 3D), decreasing from approximately 90% in the control to values close to zero at the highest concentrations.

According to the International Organization for Biological Control (IOBC) classification, in a no-choice test, EOPF treatments showed increasing toxicity with concentration in the pre-parasitism assay. Based on E_1_%, treatments ranged from slightly harmful (class 2: 2500–10,000 mg L^−1^) to moderately harmful (class 3: 20,000–40,000 mg L^−1^), while E_2_% indicated higher toxicity, with 2500–7500 mg L^−1^ classified as moderately harmful (class 3) and ≥10,000 as harmful (class 4), like Engeo Pleno^TM^ S. In the post-parasitism assay, all EOPF concentrations were classified as harmless (class 1) based on E_1_%, but E_2_% revealed moderate to high toxicity, with most concentrations in class 3 and 40,000 mg L^−1^ reaching class 4, equivalent to Engeo Pleno^TM^ S (Table 3).

In the free-choice test, EOPF significantly reduced parasitism and emergence in treated eggs at all concentrations, with parasitism ≤ 13% and emergence near zero from 5000 mg L^−1^ onward, not differing from Engeo Pleno^TM^ S. In contrast, untreated eggs were preferred, showing higher parasitism (75–88%) and emergence, although both declined with increasing EOPF concentration. Emergence in untreated eggs ranged from 71% at 2500 mg L^−1^ to near zero at 40,000 mg L^−1^, indicating a strong deterrent effect and indirect impact of OEPF on parasitoid performance (Table 4).

According to the IOBC classification, in the free-choice test, EOPF was classified as moderately harmful (class 3) to *T. podisi* on treated eggs, based on both E1% and E2%, with emergence reduction reaching class 4 (harmful) from 5000 mg L^−1^, similar to Engeo Pleno ^TM^ S. In contrast, for untreated eggs, acetone and water were classified as harmless (class 1) based on E1%, indicating low impact on parasitism preference; however, E2% revealed increasing toxicity with concentration, with treatments ≥ 5000 mg L^−1^ classified as moderately harmful (class 3) (Table 5).

## 3. Discussion

Plant-derived EOs have emerged as promising tools for pest management due to their broad biological activity and potential to reduce reliance on conventional insecticides. However, their practical incorporation into IPM depends not only on efficacy against target pests but also on their compatibility with beneficial organisms. In this context, EOPF demonstrated promising bioactivity against *E. heros*, with effects varying according to development stage and applied concentration. The EOPF exhibited pronounced ovicidal activity, particularly in early embryonic stages, and induced significant sublethal and lethal effects on nymphs, confirming its potential as an effective control agent. However, its selectivity toward the egg parasitoid *T. podisi* depended on the timing of exposure. In the pre-parasitism assay, the EOPF markedly reduced parasitism and emergence in a concentration-dependent manner, whereas in post-parasitism conditions, parasitism remained unaffected but parasitoid emergence was significantly impaired at higher concentrations. A free-choice test further revealed a strong repellent effect, with parasitoids preferentially avoiding treated eggs. Overall, although the EOPF effectively suppressed *E. heros*, its moderate to high toxicity to *T. podisi*, particularly affecting emergence, indicates potential limitations for its compatibility within IPM precepts.

The chemical profile of the EOPF revealed a complex mixture dominated by sesquiterpenes and monoterpenes, with a high proportion of identified compounds (85.37%). Several of these compounds have been reported in the literature as potential insecticides, exhibiting toxicity across different insect species and, in some cases, levels of lethality comparable to synthetics [14,17,18,19,20]. The predominance of compounds such as caryophyllene, β-eudesmene, eremophyllene, and germacrene D suggests that sesquiterpenes play a central role in the biological activity of this EO, as these compounds are widely associated with insecticidal, repellent, and growth-disrupting effects. In addition, monoterpenes such as β-ocimene and β-pinene may enhance toxicity due to their volatility and known neurotoxic action on insects [21]. Particularly noteworthy is the presence of 1-naphthalenol as one of the major components of the EOPF. This compound, along with its isomer 2-naphthalenol (detected in lower concentration), is structurally related to substances used in insecticide formulations, such as carbaryl, a carbamate that acts as an acetylcholinesterase inhibitor. Insecticides with this mode of action interfere with cholinergic signaling and neuromuscular function, leading to paralysis and mortality [22,23]. Although there are no reports of 1-naphthalenol being used directly against *E. heros*, its occurrence in EOPF suggests a possible neurotoxic mechanism contributing to the observed bioactivity [24]. Such a mechanism could represent an important advantage in resistance management, as it could provide a mode of action distinct from those of commonly used insecticides targeting stink bugs, which is a key strategy to mitigate resistance evolution through rotation schemes [25].

The EOPF exhibited pronounced ovicidal activity against *E. heros*, with egg susceptibility strongly dependent on embryonic stage. The higher inviability observed in 1- and 2-day-old eggs indicates that early embryogenesis represents a critical window of vulnerability, whereas the reduced effect in older eggs suggests increased structural and physiological resistance as development progresses. This stage-dependent pattern is consistent with studies on EO, in which egg susceptibility is closely related to developmental stage and duration of exposure [26,27]. The ovicidal effect can be explained, in part, by the lipophilic nature of EO constituents, which facilitates their penetration through the chorion and micropyles, impairing gas exchange and potentially leading to hypoxia. In addition, this property enhances the diffusion of toxic compounds into the eggs, directly affecting embryonic development [27,28,29]. Although early-stage embryos may be less immediately affected by oxygen limitations, prolonged exposure to EOPF components likely increases toxicity, contributing to the high inviability observed. Conversely, the higher hatching rates in more developed eggs may reflect shorter exposure periods and the presence of more advanced physiological defenses. Furthermore, sublethal effects observed in nymphs emerging from treated eggs may result from both chronic and acute exposure pathways. Prolonged contact with EOPF during embryogenesis can disrupt physiological processes and cuticle formation, impairing successful ecdysis [27,28], while residual EOPF on the chorion may cause acute toxicity after hatching [29]. This effect may be intensified by the gregarious behavior of *E. heros* nymphs, which remain aggregated in the eggshell, increasing their exposure to residual compounds [30].

The EOPF caused significant mortality in *E. heros* nymphs from the lowest concentration tested, confirming its high insecticidal activity across immature developmental stages. Third-instar nymphs were the most susceptible, whereas fifth-instar nymphs showed comparatively lower mortality, likely due to increased cuticular thickness and more developed detoxification mechanisms [25,31]. Adults also exhibited substantial mortality, particularly at higher concentrations, indicating that the EOPF remains effective across immature and mature stages. Its lipophilic constituents likely facilitate cuticular penetration by interacting with the waxy epicuticle, while volatilization may allow entry through spiracles, enhancing toxicity [26,27,28]. Behavioral observations during bioassays support a rapid toxic effect, as individuals exhibited immediate mortality or symptoms such as reduced mobility and leg spasms, suggesting neurotoxic action affecting the muscular and nervous systems, which are consistent with poisoning symptoms documented following exposure to EO constituents [24,32]. The rapid and significant mortality observed underscores the potential of EOPF as an effective control agent against *E. heros*. However, stage-specific differences in susceptibility emphasize the importance of optimizing application timing to maximize efficacy within IPM.

In addition to their toxicity to *E. heros*, the compounds present in EOPF affect egg parasitism by *T. podisi*. In our free-choice bioassay, the *T. podisi* females were repelled by treated eggs, resulting in a greater parasitism rate on untreated eggs, suggesting that volatile constituents of EOPF can disrupt host-associated olfactory cues and influence parasitoid choice [10]. Under field conditions, this behavior may be advantageous, as eggs not affected by the EOPF (mainly in the lower and median plant canopy section) can still be parasitized, contributing to pest suppression by preventing nymph emergence from eggs that remain viable. Moreover, during the bioassay, adult parasitoids were not killed by EOPF, remaining alive even after 24 h of exposure, which reinforces its potential compatibility at the adult stage. Regarding emergence, the results indicate the presence of toxic volatile compounds in the EO to *T. podisi*, as parasitized eggs showed reduced emergence rates. This suggests that volatile components present in the air within the experimental arena may have penetrated the eggs, negatively affecting parasitoid development, a pattern consistent with documented sublethal effects of chemical exposures on parasitoid performance [33].

The effects of EOPF on *T. podisi* were strongly dependent on the timing of exposure, with a no-choice test revealing important implications for its selectivity for the target egg parasitoid. In the pre-parasitism bioassay, the EOPF significantly reduced the parasitism and emergence rate in a concentration-dependent manner, indicating a marked negative impact on host location, acceptance, and successful development. Similar reductions in parasitism have been associated with both toxicity and sublethal effects of chemical compounds on parasitoids, including behavioral interference during host searching [33,34]. This reduction in parasitism may be explained not only by toxicity but also by disruption of key semiochemical cues, as volatile compounds can interfere with host-finding behavior. Egg recognition by *T. podisi* relies on visual and, especially, olfactory stimuli, including chemical signals emitted by host eggs [10,35]. Thus, EOPF applications may mask or disrupt these cues, ultimately reducing parasitism rates. The strong decline in emergence, reaching complete inhibition at ≥10,000 mg L^−1^, further indicates that EOPF residues can impair immature development, likely due to the penetration of volatile compounds into host eggs and subsequent effects on parasitoid offspring [33,36]. According to the IOBC classification, toxicity increased with EOPF concentration, ranging from slightly to moderately harmful based on parasitism and reaching harmful levels (class 4) for emergence, in comparison with the synthetic insecticide used as a positive control [37].

In contrast, in the post-parasitism bioassay, parasitism rates remained high across EOPF treatments, indicating that once oviposition occurs, host acceptance is no longer affected by EO presence. This is consistent with the fact that host selection in egg parasitoids is primarily mediated by olfactory and contact chemical cues prior to oviposition, whereas post-oviposition development occurs within the protected host environment [35]. However, the progressive reduction in emergence demonstrates that immature parasitoids are highly susceptible to EOPF exposure. Similar effects have been observed for other chemical compounds, in which residues or volatile fractions penetrate the egg chorion and negatively affect parasitoid development, leading to mortality before adult emergence [33,34,36]. This suggests that EOPF compounds may diffuse through the egg chorion and interfere with physiological processes during immature development. Accordingly, IOBC classification based on reduction in emergence categorized treatments as moderately to highly toxic, with higher concentrations reaching harmful levels [37].

From a practical perspective, the promising bioactivity demonstrated by EOPF provides a strong basis for further development as a botanical insecticide. As Eos are complex natural mixtures whose qualitative and quantitative composition may vary according to plant origin, environmental conditions, development stages, harvesting period, and extraction procedures, future studies aimed at chemical standardization could contribute to improving the reproducibility and consistency of EOPF-based products [38]. Based on the chemical profile, major constituents of EOPF—such as caryophyllene, β-eudesmene, 1-naphthalenol, eremophyllene, germacrene D, β-ocimene and β-pinene—may represent useful candidate chemical markers for future quality-control approaches. In addition, chromatographic fingerprinting combined with defined concentration ranges for selected compounds may help establish a more consistent relationship between chemical composition and biological activity [39,40]. Further investigation of the contribution of individual constituents and their possible synergistic interactions may also provide valuable information on the mechanisms underlying EOPF insecticidal activity. Considering the promising responses observed under laboratory conditions, formulation strategies, including emulsifiable concentrates and nanoemulsion-based systems, may further improve the dispersion, stability, persistence, and delivery of EOPF and potentially enhance its performance under more realistic conditions [41,42,43]. Semi-field and field studies would therefore represent an important next step to refine application rates, evaluate residual activity and efficacy against naturally occurring *E. heros* populations, and assess crop safety and compatibility with *T. podisi* under practical exposure scenarios. Altogether, these complementary studies may support the development of effective application strategies that preserve the insecticidal potential of EOPF while favoring its integration into soybean IPM programs.

## 4. Materials and Methods

### 4.1. Chemical Composition Analysis

The chemical composition of EOPF was analyzed by gas chromatography coupled with mass spectrometry using a Shimadzu QP2010 Plus (Shimadzu Corporation, Kyoto, Japan). Separation was achieved on an RTX-5 capillary column (30 m × 0.25 mm × 0.25 μm), using helium as the carrier gas at a constant flow rate of 1.13 mL min^−1^. The injector temperature was maintained at 220 °C, and samples were introduced under split conditions at a ratio of 1:3. The oven temperature was initially maintained at 40 °C for 10 min, increased to 220 °C at 3 °C min^−1^, and held at the final temperature for 7 min. The mass spectrometer was operated in electron ionization mode at 70 eV, with the ion-source and interface temperatures set at 250 °C. Mass spectra were acquired over an m/z range of 92. Compounds were identified by comparing their mass spectra with those of commercial libraries [44] and by their linear retention rates [45] after injection of a homologous series of alkanes (C_8_–C_26_) under the same experimental conditions, and compared with data from the literature [46].

### 4.2. Tested Insects

*Euschistus heros* and *T. podisi* populations were maintained under controlled conditions (25 ± 2 °C, 70 ± 10% relative humidity (RH), and a photoperiod of 14 L: 10 D h). Initially, *E. heros* adults were collected from soybean (31°49′06″ S, 52°31′35″ W; Pelotas, Rio Grande do Sul, Brazil) and separated by developmental stage and kept in 8 L plastic containers with airtight lids, perforated laterally and covered with voile fabric for ventilation. Individuals were provided with a natural diet consisting of bean pods, sweet potatoes, and peanuts, replaced as needed. Moisture was maintained by placing a Petri dish containing cotton soaked in distilled water at the bottom of each container. White felt strips were fixed to the inner walls of the containers with adhesive tape and served as oviposition substrate and shelter.

The parasitoid *T. podisi* was established from parasitized eggs obtained from Embrapa Soja (23°11′37″ S, 51°11′03″ S; Londrina, Paraná, Brazil). The parasitized eggs were maintained in glass tubes until adult emergence. Then, adults were transferred to glass tubes sealed with plastic film and stored in a Biochemical Oxygen Demand (BOD) chamber (at 25 ± 2 °C, 70 ± 10 RH, and a photoperiod of 14 L: 10 D h). Males and females were kept together and provided with droplets of undiluted honey applied to the inner wall of the rearing tubes as a food source. For colony maintenance, *E. heros* eggs were provided to adult *T. podisi* for parasitism every 2 days.

### 4.3. Bioassays

#### 4.3.1. Effects of EOPF on Different Embryonic Stages of *E. heros*

To assess the ovicidal action of EOPF, topical applications were performed on *E. heros* eggs aged 1, 2, 3, 4, and 5 days from the laboratory population. For each group, 10 eggs were fixed to strips of blue Canson^®^ paper (5 × 0.5 cm) with non-toxic white glue. Ten strips were used per treatment, with each strip considered an experimental unit (n = 10), totaling 100 eggs per age × EO concentration combination. Then, different concentrations of EOPF were applied topically to the cards containing the *E. heros* eggs: 2500, 5000, 7500, 10,000, 20,000, and 40,000 mg L^−1^, respectively, and two negative controls: distilled water and acetone, since the EO was diluted in PA acetone for concentration. As a positive control, the recommended rate (250 mL per 150 L of spray solution) of a commercial insecticide based on thiamethoxam + lambda-cyhalothrin (Engeo Pleno^TM^ S, Syngenta Crop Protection Ltd.a. São Paulo, Brazil) was used. The concentrations were applied to the eggs by spraying with a Vonder^®^ airbrush, calibrated to a pressure of 30 psi, with a 0.3 mm nozzle, allowing the deposition of 1.5 mg cm^−2^ of each solution/concentration. After spraying, the egg cartons were allowed to dry at room temperature. The paper cards were then stored separately in 10 mL glass tubes, capped with cotton moistened with distilled water. Hatching analysis was performed daily by counting the nymphs for 6 days after the start of hatching. After hatching, the nymphs were kept until they molted into the next instar, and the survivors were counted.

#### 4.3.2. Nymphicidal and Adulticidal Activities of EOPF on *E. heros* by Topical Application

Applications were performed on nymphs at 3rd, 4th, and 5th instars, as well as on adults of *E. heros*, with ten insects per replicate and ten replicates per treatment (n = 10), totaling 100 nymphs per treatment. Bioassays were conducted in 90 mm Petri dishes lined with paper towel disks covering the bottom surface. The EOPF was diluted in acetone to obtain concentrations of 2500; 5000; 7500; 10,000; 20,000; 40,000 mg L^−1^. Treatments were applied topically using a micropipette, delivering 1 µL per insect to the prothoracic region. Control treatments consisted of 1 µL per insect of distilled water, acetone, or the commercial insecticide Engeo Pleno^TM^ S at the recommended rate. Mortality was evaluated by recording the number of surviving insects at 24, 48, 72, and 96 h after insect exposure.

#### 4.3.3. Selectivity of EOPF on the Egg Parasitoid *T. podisi*

##### Effect on Parasitism Preference in a Free-Choice Test

The preference of treated or untreated *T. podisi* eggs of *E. heros* with EOPF was evaluated. The treatments consisted of EOPF concentrations of 2500; 5000; 7500; 10,000; 20,000; 40,000 mg L^−1^. Distilled water and acetone were used as negative controls, while the positive control consisted of the insecticide Engeo Pleno^TM^ S applied at the recommended field rate according to the Brazilian Ministry of Agriculture and Livestock [47]. Egg masses were sprayed using a Vonder^®^ airbrush calibrated at 30 psi, delivering a deposition rate of 1.5 mg cm^−2^ per treatment. For each concentration, ten replicates were prepared, each consisting of ten eggs affixed to blue Canson^®^ paper. After application, the treated eggs were allowed to air-dry at room temperature. Each replicate consisted of a pair of egg cards (one treated and one untreated) placed inside a glass tube. A single 72 h-old female *T. podisi* was introduced and allowed to parasitize the eggs for 24 h. After this period, the female was removed, and the eggs were maintained under controlled conditions (25 ± 2 °C, 70 ± 10 RH, and a photoperiod of 14 L: 10 D h). Parasitism was assessed at 24 h intervals by monitoring parasitoid development until 11 days after exposure, when adult emergence began [9].

##### Effect of EOPF on Pre- and Post-Parasitism of *T. podisi*

In the pre-parasitism assay, 24 h-old eggs of *E. heros* were sprayed with EOPF at concentrations of 2500 to 40,000 mg L^−1^. Distilled water and acetone were used as negative controls, and Engeo Pleno^TM^ S, applied at the recommended rate, was used as the positive control. Applications were performed using a Vonder^®^ airbrush operated at 30 psi, 0.3 mm nozzle, 1.5 mg cm^−2^. Ten replicates were prepared per treatment, each consisting of ten *E. heros* eggs mounted on blue Canson^®^ paper. After treatment, the egg cards were air-dried and individually placed in glass tubes containing one 72 h-old *T. podisi* female. After 24 h of exposure, the females were removed, and the eggs were incubated in a BOD chamber at 25 ± 2 °C, 70 ± 10 RH, and a photoperiod of 14:10 h (L:D). Evaluations were performed daily for up to 11 days [9]. Parasitism was calculated for each replicate as the percentage of parasitized eggs relative to the total number of eggs offered, whereas adult emergence was calculated as the percentage of emerged parasitoids relative to the total number of parasitized eggs.

In the post-parasitism assay, ten replicates were conducted under the same experimental conditions. Egg masses of *E. heros* were first exposed to *T. podisi* females for 24 h. After this period, the females were removed, and the parasitized eggs were sprayed with EOPF solutions diluted in acetone. The treated eggs were then incubated for 11 days under the same controlled conditions, and parasitism and adult emergence were evaluated at 24 h intervals [9].

#### 4.3.4. Classification of Selectivity

To classify the EOPF impact on *T. podisi*, the mean number of parasitized eggs per female was calculated for each treatment. The percentage reduction in parasitism and adult emergence was determined relative to the negative control (distilled water) using the equation: E (%) = (1 − Vt/Vc) × 100, where E_1_ represents the reduction in parasitism and E_2_ the reduction in emergence, Vt is the mean value observed in the treatment, and Vc is the mean value in the control. Based on these values, treatments were classified according to IOBC standards as follows: class 1, harmless (E < 30%); class 2, slightly harmful (30% ≤ E ≤ 79%); class 3, moderately harmful (80% ≤ E ≤ 99%); and class 4, harmful (E > 99%) [37].

### 4.4. Data Analysis

For analysis of data from laboratory bioassays, linear, quadratic, or exponential regressions were fitted to the data related to factors of quantitative levels, depending on the behavior of the data in relation to the levels, and the accuracy of adjustment was assessed. For data from factors of qualitative levels, generalized linear models were used. Model adequacy was assessed with half-normal plots with simulation envelopes using the hnp package (Yers 2008), [48]. Differences between treatments were determined using Tukey’s post hoc test (*p* < 0.05) with adjusted *p*-values via the “glht” function from the multcomp package [49]. Lethal concentrations required to kill 50% (LC_50_) and 90% (LC_90_) of *E. heros* were estimated using a Probit model implemented in R statistical software version 4.4.3 [50], following Finney [50].

## 5. Conclusions

The EOPF is a chemically complex mixture dominated by sesquiterpenes and monoterpenes, including caryophyllene, β-eudesmene, 1-naphthalenol, eremophyllene, germacrene D, β-ocimene, and β-pinene, which likely drive its potent bioactivity. EOPF exhibited ovicidal, nymphicidal, and adulticidal effects against *E. heros*, with early-stage eggs and third-instar nymphs being the most susceptible, highlighting a clear stage-dependent vulnerability. EOPF also impacted egg parasitism by *T. podisi*, with pre-parasitism exposure reducing both parasitism and adult emergence, whereas post-parasitism exposure mainly impaired parasitoid development and emergence. In the free-choice assay, females preferentially parasitized untreated samples, indicating a deterrent effect that may reduce parasitoid performance in treated environments. Overall, EOPF represents a promising botanical insecticide candidate for *E. heros* management; however, its compatibility with *T. podisi* and its integration into soybean IPM programs require further evaluation, particularly regarding concentration, application timing, formulation, and exposure under field conditions.

## Figures and Tables

**Figure 1:**
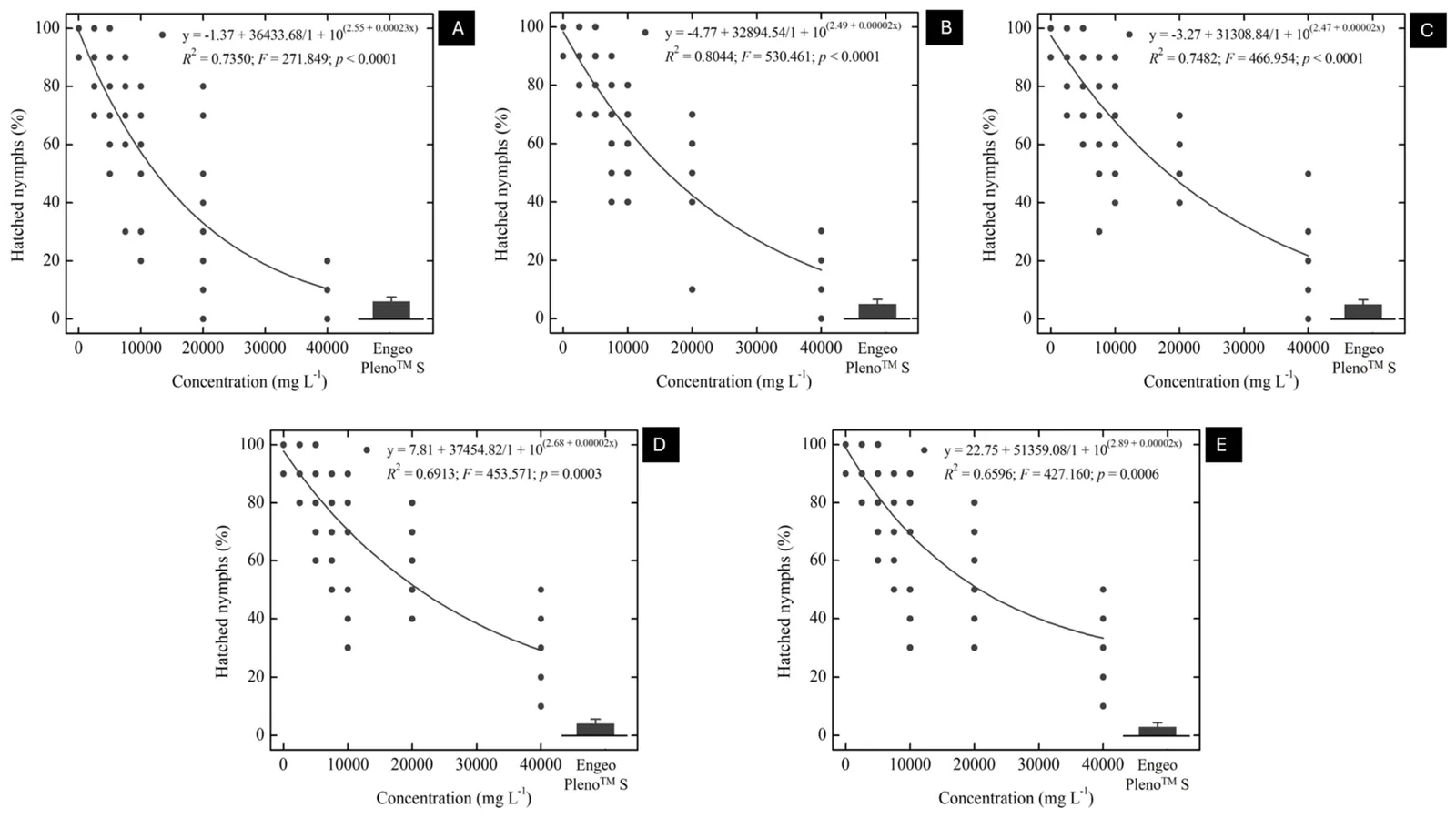
Ovicidal activity of *Piper fuligineum* essential oil (EOPF) against *Euschistus heros* eggs of different ages. Eggs from a laboratory population were treated by application at (**A**) 1, (**B**) 2, (**C**) 3, (**D**) 4, and (**E**) 5 days of age. The percentage of hatched nymphs was evaluated across increasing EOPF concentrations (mg L^−1^). Points indicate observed values, whereas solid lines represent the fitted concentration–response models. Engeo Pleno^TM^ S was used as a positive control.

**Figure 2:**
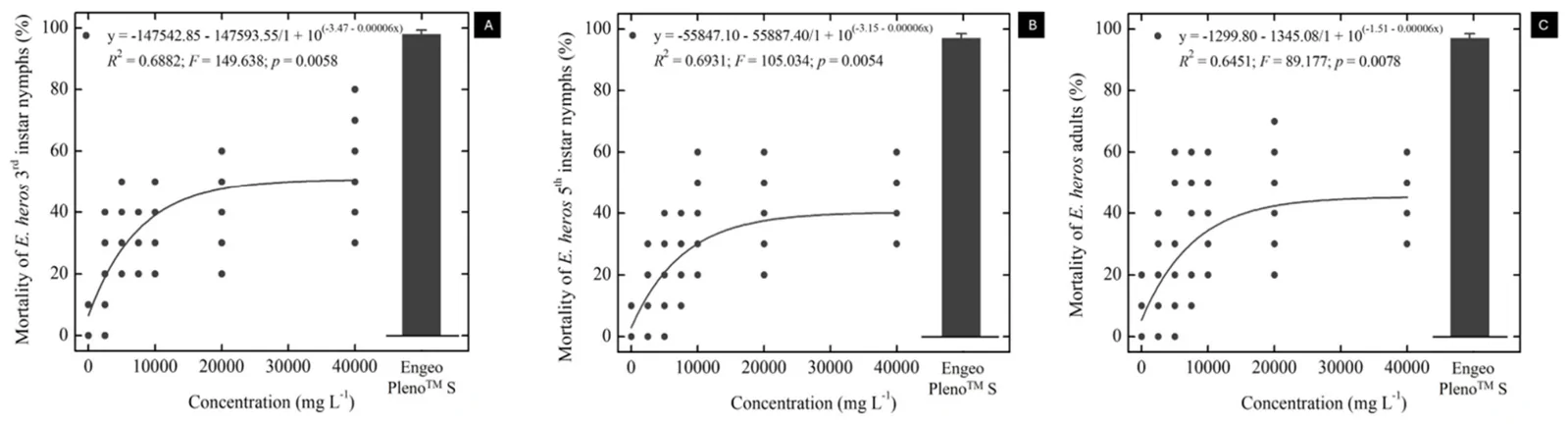
Mortality of third-instar nymphs (**A**), fifth-instar nymphs (**B**), and adults (**C**) of *Euschistus heros* exposed to different concentrations of *Piper fuligineum* essential oil (mg L^−1^). Points represent the observed mortality values, whereas solid lines represent the fitted concentration–response models. Engeo Pleno^TM^ S was used as a positive control.

**Figure 3:**
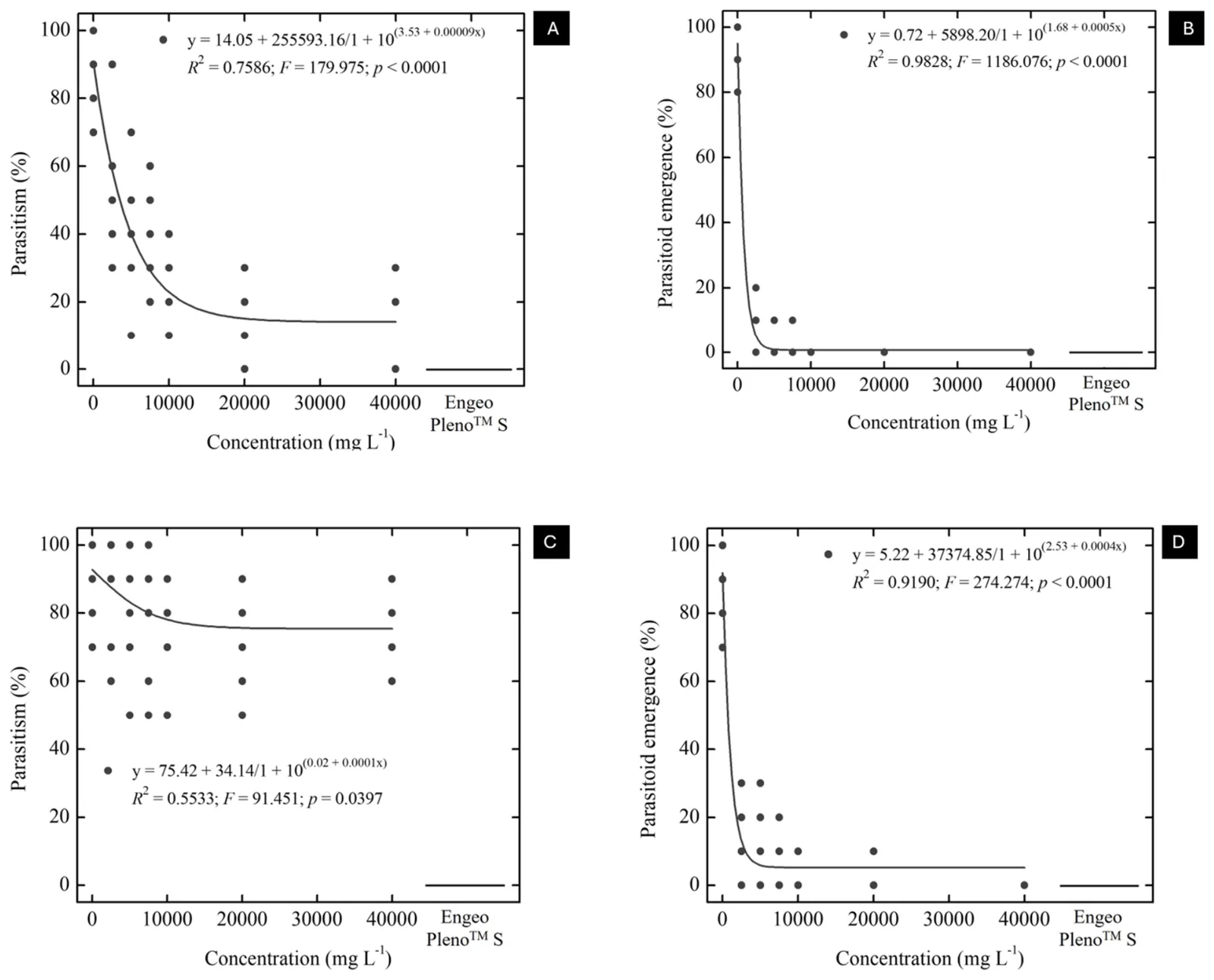
Effects of concentrations of *Piper fuligineum* essential oil on the parasitism and emergence of *Telenomus podisi* under pre- and post-parasitism bioassay exposure conditions. (**A**) parasitism and (**B**) parasitoid emergence in the pre-parasitism; (**C**) parasitism and (**D**) parasitoid emergence in the post-parasitism. Points indicate observed values, and solid lines represent the fitted concentration–response models. Engeo Pleno^TM^ S was used as a positive control.

**Table 1:** Chemical composition of the essential oil of *Piper fuligineum* determined by gas chromatography-mass spectrometry.

Retention Time	%	Compound	Molecular Structure
3280	4.41	Propyl Ethanoate	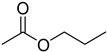
14,339	1.87	α-Pinene	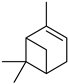
16,981	5.16	β-Pinene	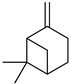
21,378	1.92	Trans-β-Ocimene	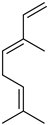
22,004	5.48	β-Ocimene	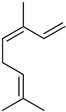
39,036	2.70	α-Copaene	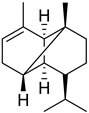
40,776	10.60	Caryophyllene	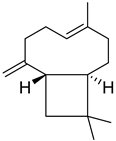
43,207	2.62	α-Selinene	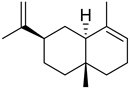
43,327	7.40	Germacrene D	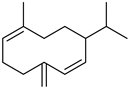
43,526	9.09	Eremofilene	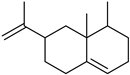
43,975	10.46	β-Eudesmene	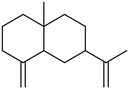
44,649	1.93	α-Farnesene	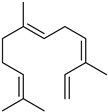
45,112	1.29	Isoledene	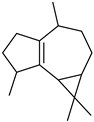
46,658	2.02	trans-Nerolidol	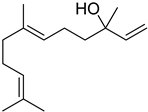
46,868	2.07	(-)-Spathulenol	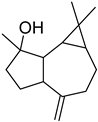
47,062	2.05	β-Caryophyllene oxide	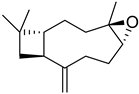
48,329	3.03	Globulol	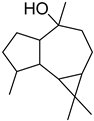
49,764	9.88	1-Naphthalenol	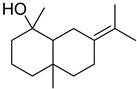
55,782	1.39	2-Naphthalenol	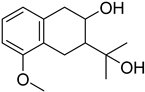

**Table 2:** Estimated LC_50_ and LC_90_ values for eggs, third- and fifth-instar nymphs, and adults of *Euschistus heros* exposed to *Piper fuligineum* essential oil by topical application.

Phase	Age	N^o a^	Slope (±EP)	CL_50_ (±IC95) ^b^	CL_90_ (±IC95) ^c^	χ^2 d^
Egg	24 h	100	2.03 ± 0.15	1100 (900–1300)	4700 (3500–7500)	2.83
Egg	48 h	100	1.89 ± 0.21	1400 (1100–1700)	6800 (4700–11,000)	2.84
Egg	72 h	100	1.73 ± 0.21	1600 (1300–2100)	9000 (5800–17,000)	2.96
Egg	96 h	100	1.74 ± 0.22	1900 (1600–2600)	10,800 (6800–22,000)	3.14
Egg	120 h	100	1.55 ± 0.21	2000 (1600–2800)	13,600 (7900–33,400)	4.02
Nymph	3rd inst.	100	0.70 ± 0.05	3000 (2300–3800)	20,000 (15,000–41,000)	5.68
Nymph	5th inst.	100	0.68 ± 0.09	9700 (4400–27,200)	54,800 (22,000–158,300)	4.91
Adult	-	100	0.70 ± 0.10	6200 (3300–9100)	30,900 (16,000–242,600)	5.43

^a^ N^o^: number of *E. heros* individuals tested; ^b^ LC_50_: lethal concentration required to cause 50% mortality; ^c^ LC_90_: lethal concentration necessary to cause 90% mortality; CI: 95% confidence interval; ^d^ χ^2^: chi-square value.

**Table 3:** International Organization for Biological Control toxicity classification of *Piper fuligineum* oil to *Telenomus podisi* based on reductions in parasitism and adult emergence in pre- and post-parasitism no-choice test.

Treatments	Pre-Parasitism				Post-Parasitism			
	E_1_% ^a^	Class.	E_2_% ^b^	Class.	E_1_% ^a^	Class.	E_2_% ^b^	Class.
Water	-	-	-	-	-	-	-	-
Acetone	7.00	1	8.00	1	7.00	1	8.00	1
2500	44.33	2	94.73	3	2.06	1	87.37	3
5000	61.85	2	96.84	3	4.12	1	87.36	3
7500	61.85	2	98.94	3	2.06	1	92.63	3
10,000	76.28	2	100	4	4.12	1	94.73	3
20,000	81.44	3	100	4	6.19	1	95.70	3
40,000	90.72	3	100	4	2.15	1	100	4
Engeo Pleno^TM^ S	100	4	100	4	100	4	100	4

^a^ E_1_% is the percentage reduction in parasitism; ^b^ E_2_% is the percentage reduction in emergence; Class.: 1 = harmless (E < 30%), 2 = slightly harmful (E 30 ≤ e ≤ 79%), 3 = moderately harmful (80 ≤ E ≤ 99%), 4 = harmful (E > 99%).

**Table 4:** Effects of *Piper fuligineum* essential oil on parasitism and adult emergence of *Telenomus podisi* from treated and untreated *Euschistus heros* eggs in a free-choice test.

Treatments		Treated	Untreated	
	Parasitized Eggs (%)	Emerged Parasitoids (%)	Parasitized Eggs (%)	Emerged Parasitoids (%)
Water	97.0 ± 0.15 a ^1^	95.0 ± 0.22 a	97.0 ± 0.15 a	95.0 ± 0.22 a
Acetone	93.0 ± 0.33 a	92.0 ± 0.36 a	93.0 ± 0.34 a	92.0 ± 0.36 a
2500	13.0 ± 0.50 b	2.0 ± 0.13 b	88.0 ± 0.46 a	71.0 ± 0.64 b
5000	10.0 ± 0.33 b	0.0 ± 0.00 b	83.0 ± 0.52 b	17.0 ± 0.45 c
7500	9.0 ± 0.35 b	0.0 ± 0.00 b	81.0 ± 0.41 b	11.0 ± 0.23 c
10,000	6.0 ± 0.22 b	0.0 ± 0.00 b	80.0 ± 0.47 b	7.0 ± 0.26 c
20,000	7.0 ± 0.26 b	0.0 ± 0.00 b	77.0 ± 0.50 b	9.0 ± 0.23 c
40,000	5.0 ± 0.16 b	0.0 ± 0.00 b	75.0 ± 0.56 b	0.10 ± 0.10 d
Engeo pleno^TM^ S	0.0 ± 0.00 b	0.0 ± 0.00 b	0.0 ± 0.00 c	0.0 ± 0.00 d
*F*-value	179.9043 **	773.6186 **	51.4016 **	148.2466 **
*p*-value	<0.0001	<0.0001	<0.0001	<0.0001

** significant at the 1% probability level (*p* < 0.01). ^1^ Averages followed by the same letters in the column do not differ according to the Tukey test (5%).

**Table 5:** International Organization for Biological Control toxicity classification of *Piper fuligineum* oil to *Telenomus podisi* based on reductions in parasitism and adult emergence from treated and untreated *Euschistus heros* eggs in a free-choice test.

Treatments	Treated				Untreated			
	E_1_%	Class.	E_2_%	Class.	E_1_%	Class.	E_2_%	Class.
Water	-	-	-	-	-	-	-	-
Acetone	7.00	1	8.00	1	7.00	1	8.00	1
2500	86.59	3	97.89	3	9.27	1	25,26	1
5000	89.70	3	100	4	16.49	1	82.10	3
7500	90.72	3	100	4	11.34	1	88.42	3
10,000	93.81	3	100	4	17.52	1	92.63	3
20,000	92.78	3	100	4	20.61	1	90.53	3
40,000	94.89	3	100	4	22.68	1	98.94	3
Engeo pleno^TM^ S	100	4	100	4	100	4	100	4

E_1_% is the percentage reduction in parasitism; E_2_% is the percentage reduction in emergence; Class.: 1 = harmless (E < 30%), 2 = slightly harmful (E 30 ≤ e ≤ 79%), 3 = moderately harmful (80 ≤ E ≤ 99%), 4 = harmful (E > 99%).

## Data Availability

The original data are included in the paper. Further inquiries can be directed to the corresponding author.

## Abbreviations

The following abbreviations are used in this manuscript:

EOs	Essential Oils
EOPF	*Piper fuligineum* Essential Oil
IPM	Integrated Pest Management
BOD	Biochemical Oxygen Demand
RH	Relative Humidity
IOBC	International Organization for Biological Control
